# Quality of life and unmet needs in patients with fabry disease: a qualitative study

**DOI:** 10.1186/s13023-024-03412-6

**Published:** 2024-10-18

**Authors:** Montserrat Morales, Jordi Cruz, Eduardo Brignani, Laura Acuña, Esther Lázaro, Cristina Soria

**Affiliations:** 1 Hospital Universitario 12 de Octubre, Unidad de Adultos de Enfermedades Raras y Errores Congénitos Del Metabolismo, Madrid, España; 2 Asociación MPS-LISOSOMALES, Igualada, España; 3 Departamento Médico, Chiesi, España; 4 Facultad de Ciencias de la Salud, Universidad Internacional de Valencia, Valencia, España; 5 Psicología de la Salud, Suportias, Madrid, España

**Keywords:** Quality of life, Fabry disease, Qualitative study, Unmet needs

## Abstract

**Background:**

Patients with Fabry disease (FD) consider their quality of life to be significantly affected. The majority of studies evaluate the quality of life using quantitative measures and standardised scales that offer relevant information about experience with the disease in multiple aspects. The main objective of the research was to examine in depth the quality of life and unmet needs of patients diagnosed with FD in relation to their disease and treatment. A qualitative and transversal study was carried out in two stages: (a) nine semi-structured qualitative interviews with patients and one representative of the patient association, conducted individually by phone; (b) a focus group was set up with three patients diagnosed with FD and one relative. A deductive, thematic analysis approach was used for data coding and analysis.

**Results:**

The analysis of the interviews revealed various relevant themes: experience with the disease, impact on daily activities, experience of the family and work environment, experience related to treatment and healthcare professionals, and unmet support needs. Diagnosis has a significant impact on both those suffering from the disease and on the family environment. The symptoms and evolution of the disease are highly variable among the patients interviewed and depend on the years diagnosed as well as the time taken to receive the diagnosis. The families of the interviewees have to go through an adjustment process in light of the significant psychological impact brought about by the disease. Patients show various unmet needs. The need mentioned most is to have more information, support, and understanding from people around them and society, improving empathy and raising awareness about the difficulties faced by people with FD while giving the disease visibility. A lack of social understanding is highlighted as one of the main challenges, as this does not only affect the emotional management of the disease but also has repercussions on working life and social relationships.

**Conclusions:**

It seems necessary to define possible strategies that help to improve the quality of life of patients and their experience with the disease. Some recommendations obtained from the study include: facilitate access to mental health professionals for patients and their families; improve training for specialists and coordination among them; and carry out actions to raise awareness of the disease that are aimed at the general public, the patients themselves, and the people around them.

**Supplementary Information:**

The online version contains supplementary material available at 10.1186/s13023-024-03412-6.

## Introduction

Fabry disease (FD) is a rare X-linked lysosomal storage disorder characterized by deficiency of the lysosomal enzyme α-galactosidase A (α-Gal A, E.C. 3.2.1.22), which causes a systemic accumulation of globotriaosylceramide (Gb3) and related glycosphingolipids in lysosomes in cells throughout the body. The prevalence is estimated to be 1 per 40,000–170,000 live births. The incidence of this rare disease is underestimated due to delayed diagnosis [1, 2]. Two types of FD can be distinguished: the classical form, which is the most severe phenotype due to very low or absent α-galactosidase A activity with the onset of symptoms during childhood and predominantly affecting men and the non-classical form, also referred to as late-onset, characterized by residual enzyme activity and presentation during adulthood. Women can be as severely affected as male patients with classic FD, but most of them have a more variable and attenuated phenotype. This is why they are usually categorized as non-classical patients [3].

The initial symptoms in both male and female patients affected by the classical form include angiokeratoma, anhidrosis (absence of sweating), neuropathic pain, cornea *verticillata* (gray or golden deposits in the epithelium) and gastrointestinal symptoms. As the disease evolves, progressive kidney failure, hypertrophic cardiomyopathy, cardiac rhythm disturbances, and strokes may develop. In male patients with the non-classical form and in most women, a more attenuated and variable course of the disease is observed. The life expectancy and morbidity of patients are significantly associated with the degree of damage to the affected organs [4–8].

Although enzyme replacement therapy currently exists and has shown beneficial effects on neuropathic pain, heart mass, and kidney function, complications of the disease can still occur [9, 10]. In this sense, patients suffering from this disease have a lower quality of life compared to healthy individuals, especially related to neuropathic pain and anhidrosis. These results are observed both with general and disease-specific quality of life questionnaires [11]. On the other hand, enzyme replacement therapy has a positive effect on quality of life [12, 13].

The majority of studies involving patients diagnosed with Fabry disease evaluate quality of life using quantitative measures and standardized scales that offer relevant information about experience with the disease in multiple aspects [11]. However, complementing this knowledge with the use of qualitative methodologies would help to further explore the specific needs and concerns of this group of patients, their relatives, and, consequently, the socio-health agents involved in the care administered for this disease. To our knowledge, there are very few studies focused on patients with Fabry disease and their relatives that explore their quality of life from a qualitative perspective [14–16]. The main objective of the research was to thoroughly examine the quality of life and the unmet needs of patients diagnosed with Fabry disease in Spain in relation to their disease and treatment.

## Methods

### Sample

A qualitative and cross-sectional study was carried out. The study focused on thoroughly exploring the experiences regarding the quality of life and unmet needs of patients diagnosed with Fabry disease in relation to their disease and treatment. A family member and a representative of the patient association were also included to complement the information collected from patients.

The participants were selected according to the inclusion criteria, and they were not randomly selected. The sample represents different sociodemographic profiles of patients to capture the diversity of experiences of Fabry patients. Different patient profiles were gathered based on sex, age, time of diagnosis, age at which they received the diagnosis, and geographical origin. The number of focus groups and interviews was chosen to achieve content saturation. This study was conducted in two stages. The first stage consisted of 10 semi-structured qualitative interviews (9 with patients and 1 with a patient association representative). The interviews were carried out individually by phone by a health psychologist with experience in qualitative research and rare diseases. For the second part, a focus group was set up with three patients diagnosed with Fabry disease and one relative, using joint reflection to understand the needs of patients, possible content of interest for this group of patients, and relevant aspects obtained in the individual interview stage.

The following inclusion criteria were established: Patients over 18 years of age, diagnosed with Fabry disease, with availability to conduct a telephone interview; that provided informed consent to participate in the qualitative study and to the interview being recorded for analysis purposes. For the focus group, participants were asked to have a device with a camera and microphone and an internet connection to be able to attend the meeting via the communication platform Microsoft Teams. Everyone signed a consent form for the meeting to be recorded for analysis purposes. Anyone with limitations preventing them from collaborating in the study (mental disorders, non-collaboration, limited language comprehension) were excluded.

### Data collection and analysis

All participants in the study were recruited in collaboration with the Spanish Association for Lysosomal Diseases (Asociación MPS-LISOSOMALES). The participants were informed that their participation was voluntary and free, and that they could leave the evaluation whenever they wanted, for any reason, and without any consequences. There was no material or financial remuneration for participating in the evaluation. As it was a qualitative study, the patients were not required to take any other medication, undergo any specific examinations, or attend other visits, apart from the usual ones indicated by their doctor. No changes were made to their ongoing treatment or the care they were receiving.

This study was carried out by means of semi-structured qualitative interviews, conducted individually by phone by a health psychologist with experience in qualitative research and rare diseases. A semi-structured interview guide was used during the interviews to raise pertinent open-ended questions for the participants to address. The structure and questions of the interview were developed by three psychologists, and were organised around 5 areas: experience with the disease; experience of the family environment; experience with pharmacological treatment; experience with the team of healthcare professionals; and unmet support needs. The interviews were carried out between November and December 2022, with an approximate duration of 60 min per interview. The focus group lasted for two and a half hours and was conducted by a health psychologist using the same semi-structured guide. All participants gave their consent to participate in the study. Data from the focus group and interviews were transcribed, and the accuracy of the interview transcripts generated was verified by the authors.

A deductive approach was used for data coding. and a top-down approach was adopted for analysis, whereby the researcher introduced a series of established concepts or topics that they used to code and interpret the data. A six-phase approach to thematic analysis was followed [17], including: Phase (1) Familiarising yourself with the data; Phase (2) Generating initial codes; Phase (3) Searching for themes; Phase (4) Reviewing potential themes; Phase (5) Defining and naming themes; Phase (6) Producing the report.

### Rigor of the study

Several strategies were used to achieve reliability, validity, and generalisability of the study findings [18]. A coding system for research was developed through semi-structured interviews. This coding system ensures that the meaning of the analysis is the same between coders while improving the validity and certainty of the findings. In addition, the expressions and comments in free format of the participants were taken into account as additional data for the questions of the semi-structured interviews, thus considering the unusual cases in comparison with the most common cases. Researcher bias was controlled by the participation of several researchers, one of whom was dedicated to conducting interviews, and three researchers participated separately in the analysis of the interviews. The participation of different researchers enhances the triangulation of the study and gives it greater validity. In addition, a methodological triangulation was also carried out by obtaining the data in two ways: through individual interviews and through focus groups.

## Results

The main results obtained from the testimonies collected in the interviews are structured and described in the next section. See Table 1 for a greater detail of the quotes of the participants through the interviews and the focus group.

**Table 1:** Examples of the quotes of the participants

Symptomatology and disease progression	“I was always tired; I was always in pain; my back was killing me, my joints were horrible.” (Focus group). “The pain is sharper in my hands (…) in my feet; now it’s a burning sensation that I’d never felt before (…) practically 24 hours a day.” (Individual interview). “They did a lot of tests, a lot, and nothing came out. (…) There came a time when I was already taking it as practically normal.” (Individual interview). “I’ve known my father to be sick in bed all my life. (…) My classmates recalled my mother saying, ‘He’s sick; he has a high fever’.” (Individual interview).
Medical specialists visited	*“I was told that the boy was fine*,* that the problem wasn’t his*,* but mine*,* a psychological problem*,* that it was me who was ill*,* and that she couldn’t help me because she was a paediatrician*,* so (…) should call security.” (Individual interview).* *“It came as a real blow. (…) The doctor just told me that it was a major disease and that I wouldn’t be able to ignore it.” (Focus group).* *“That’s why we had to do a study on the children (…) above all*,* because the result took a very long time to come (…) because of that waiting and that anguish that my children might have the disease.” (Individual interview).* *“Thanks to family screening*,* my children are in control every day. To the smallest detail*,* you can already start putting means in place*,* and you have the knowledge.” (Focus group).*
Emotional impact	*“I had an anxiety-depressive crisis because I was also the one who was carrying everything.” (Individual interview).* *“I have very severe depression (…) I don’t have a 100% normal life like other people (…) my mood is not like other people’s (…) it is a state of apathy*,* of sadness (…) of not being at ease and of feeling empty (…) of not being able to do everyday things (…) I have no desire*,* I have no desire to do those things. If I do them*,* then they don’t satisfy me either.” (Individual interview).* *“You feel lonely.” (Focus group).* *“I had severe depression; between the lack of psychologists and the fact of having an illness (…) to look young*,* to have studied (…) I became a piece of furniture.” (Focus group).* *“You have to tell your girlfriend*,* ‘Well*,* look*,* baby*,* we can’t go out tonight.’ If that person doesn’t understand you*,* then it’s very*,* very difficult to live with that.” (Individual interview).*
Resources used to cope with the disease	*“The support of the group*,* especially at the beginning (…) they open your eyes a little*,* you see what the issue is really like*,* because in principle you don’t know anyone*,* your doctor and that’s it. (…) At the meetings everyone brings forward their personal experience*,* (…) it is a relief to hear what it’s like*,* that you are learning (…) more accompanied. You are not alone*,* and that has been thanks to the groups.” (Individual interview).* *“It has been helpful for me to have a group that offers me information and contacts. I have had veral individual sessions over the phone with the group’s psychologist.” (Individual interview).* *“You realise that you are not the only one who is like this*,* and you relate to people because they are like you; (…) you talk to them*,* you have a conversation; (…) it’s not that it calms you down*,* but well*,* you think well; it’s the disease; (…) you feel understood.” (Individual interview).* *“We have to live*,* and that’s it. Live the best we can in the little or long time we have.” (Individual interview).* *“Because you already know what you have; (…) it has a name; there is an explanation for what is happening to you; (…) that is a relief on the one hand.” (Individual interview).* *“I try to lead a normal life*,* although it’s harder if I feel pain. What limits me most is diarrhoea*,* which affects me at work and in social activities.” (Individual interview).* *“I live a normal life. Any day I feel worse*,* I stay in bed.” (Individual interview).*
Impact on daily activities	*“I can’t drink because of my kidneys. (…) When everyone is dancing and drinking (…) I take three little steps and say*,* ‘Boy*,* sit down; your heart won’t let you’ (…) It affects you because you can’t share all the things you want.” (Individual interview).* *“I like activities such as hiking*,* but I need to go at a different pace because of tiredness.” (Individual interview).* *“I don’t feel like doing things (…) if I went out*,* I would spend the day on the sofa*,* or from the bed to the sofa; from the sofa to the bed*,* my husband has to persuade me a lot*,* saying*,* ‘Come on*,* let’s go out?’.” (Individual interview).* *“They can’t run*,* they can’t exercise*,* so let them do it*,* let them go out*,* and let the teacher know that the moment they feel pain*,* they have to stop.” (Individual interview).* *“The teacher forced you and you forced your body in such a way that (…) we didn’t sweat*,* because our body heated up and it had to stop somehow*,* or you would get a very high fever…” (Individual interview).*
Impact on work activities	*“The most complicated things are the symptoms (diarrhoea) and taking time off from work to go to the treatment sessions or to the doctor. (…) From time to time he reproaches him (the manager)*,* especially (…) absences for going to the doctor.” (Individual interview).* *“The diagnosis did not affect me practically*,* but above all*,* when I saw that I could no longer work because I was not concentrating*,* because I was always tired*,* because my disability was approved at first*,* the full one and yet I was not happy (…) I felt useless (…) I fell into pretty severe*,* pretty deep depression*,* which cost me my marriage.” (Individual interview).* *“Slowness affects me at work (…) I notice it more*,* I’m slower.” (Individual interview).* *“You don’t take it so seriously (…) because if you see me going to work smiling and I’m happy*,* considering what I’ve got*,* it’s not so important.” (Focus group).* *“Some colleagues take it better*,* and some take it worse. Well*,* a colleague asked me why I was going to the doctor’s so often*,* so I said I’m going to tell him*,* I’ve been diagnosed with a rare disease*,* and I have to go for treatment every fortnight. Then the next morning he says to me*,* ‘Good morning weirdo’.” (Focus group).* *“I’m retired. (…) For me*,* it was the worst because*,* now I’m not able to pick up stones*,* alright*,* but being in a school making photocopies*,* well*,* that sounds fine*,* but I don’t understand it.” (Focus group)* *“If outdoor meetings are held in the summer*,* they are adapted to take place at times when it is not as hot.” (Individual interview).* *“I had some excellent colleagues; (…) I wasn’t going to work for the day; (…) they would let me go in the afternoon.” (Individual interview).* *“Sometimes*,* you are more tired than usual; there are days that maybe you can’t even get out of bed*,* but (…) I have been highly respected in this regard; I have fairly suitable working hours so that I can go out on the days that I have to go to the hospital.” (Individual interview).*
Experience of the family environment	*“So what happens to them affects you as a direct relative*,* and you are living with a person who you also know has a disease …that is degenerative and has no cure; you live with it as a family too. So you suffer it in a different way*,* but you do suffer it.” (Focus group).* *“Those who live with an ill person have to be flexible*,* adapt*,* and really accept their needs.” (Individual interview).* *“As a father*,* (…) you process the consequences and the worry.” (Focus group).* *“When a mother is told that her son was not well*,* it was because he was not well*,* no mother wants to take her child to the hospital every day. (…)I spent the nights awake because I was doing nothing but mulling it over in my head because everyone told me that it was my fault.” (Individual interview).* *“At the hospital*,* they told me to wait to see if the genetic tests came out*,* and (…) in other places*,* they told me*,* ‘Well*,* take a chance’ (…) in the end*,* I did nothing; I didn’t have any children.” (Individual interview).* *“That’s why I got divorced. (…) I was told*,* ‘Well*,* when you come home*,* you’re always tired*,* you’re always in pain*,* you’re always knackered’.” (Individual interview).* *“It’s really difficult to explain (…) because people just can’t understand it*,* (…) physically*,* you look fine.” (Individual interview).* *“Although he knows perfectly well what I have*,* (…) we also don’t try to talk much about how I’m doing.” (Individual interview).* *“The disease was like a taboo when it started; I’ve had to hear things from my family like them blaming me*,* and some part of the family hasn’t wanted to know anything either.” (Individual interview).*
Experience with pharmacological treatment	*“I found it really difficult to start treatment; I mean*,* I’ve had to fight quite hard for it… and a lot of turning up at the hospital*,* putting a bit of pressure on the managers*,* on the hospital*,* and why not*,* and why this*,* and why that “It’s really expensive; we have to increase the budget.” (Individual interview).* *“I know there have been issues in some Spanish autonomous regions for some Fabry patients to receive enzyme replacement treatment.” (Individual interview).* *“I don’t notice that I feel good or bad*,* but it’s true that I am fine; in other words*,* I’m not worse.” (Individual interview).* *“I noticed it as soon as they started to give it to me*,* many years ago*,* I noticed that I felt better*,* I got less tired*,* and I could do more things*,* but right now with every dose they give me*,* I don’t notice anything.” (Focus group).* *“I have started to sweat a little from my armpit*,* and for me*,* this is really something*,* because I had never felt sweat.” (Focus group).* *“My family and I*,* were there from 8 in the morning until 7 in the evening on the first day (at the hospital).” (Focus group).* *“My brother and I*,* as we have to be given the dose*,* or rather*,* as the medication is so expensive*,* what is left over from one of us is given to the other*,* (…) so we both have to go together*,* (…) and I have to organise myself with my brother no matter what.” (Focus group).* *“I am calmer at the hospital because of the possibility of being cared for by the staff if any problems arise.” (Individual interview).* *”We were so scared that it would give us an adverse reaction (…) that if we hadn’t been at the hospital*,* I don’t know what I would have done.” (Individual interview).*
Team of health professionals	*“I had to go for an analysis at least once a year and get a series of tests done yearly because this is a disease that needs a lot of specialists working on it and nobody takes responsibility for us.” (Individual interview).* *“The care could be a little better for internal medicine because I’ve had problems with him; (…) he told me that women don’t suffer from this disease.” (Individual interview).* *“There is no one to coordinate with each other.” (Focus group).* *“I’ve had to look for a psychiatrist (…)*,* you have to pay the psychologist yourself if you need this support*,* and I’m telling you that we need this support*,* and I’m sure that it’s not just our disease*,* but any rare disease for which there is no cure.” (Individual interview).* *“Sharing your own experience with that of other people*,* being able to meet up one day*,* having a meal together*,* and sharing information is unprecedented; I mean*,* it gives you more peace of mind; (…) you’re not alone.” (Individual interview).* *“The faith in knowing that they’re there*,* in knowing that they are working*,* not just for me but for many other ill people*,* of course*,* with other rare diseases (…) it always gives me peace of mind*,* makes me feel calm*,* and gives me confidence.” (Individual interview).*
Unmet support needs	*“These patients (…) should retire earlier (…) because some run the risk of being fired and having nothing*,* and others run the risk of not finding a job.” (Individual interview).* *“I think there are more people with Fabry than those who are actually diagnosed (…) people who have heart and kidney problems (…).” (Individual interview).* *“The loss of interest from the internist once some time had passed from the diagnosis*,* despite the fact that he was very involved at first.” (Individual interview).* *“They should take us a little seriously because there are doctors who take it a little bit far.” (Individual interview).* *“There are many doctors who do not know how to assess Fabry disease and also do not let or do not like you to explain what the disease is.” (Individual interview).* *“Enzyme replacement therapy works well if all the support given to the patient is adequate. It is important that the patient feels better*,* is better accompanied*,* and has that help from physiotherapy*,* a psychologist or whatever care they need*,* support in their day-to-day lives (…) that patient will be in a better condition.” (Individual interview).* *“I know that there have been problems in some communities for some Fabry patients to receive enzyme replacement treatment.” (Individual interview).* *“This is really important to me*,* feeling understood*,* but by people around me in general*,* society*,* not just a psychologist*,* but society in general*,* your friend*,* your work colleague*,* your neighbour.” (Individual interview).* *“The geneticist is in charge of coordinating the family tree (…) the newborn screening (…) because they are diseases that have therapeutic options and rapid action will change the course of the disease (…)*,* because today it is coordinated by the internist or the paediatrician or whoever looks after the patient*,* of course*,* but it would not be their responsibility.” (Individual interview).* *“That way of acting needs to be automatic as soon as that information or that diagnosis comes out to facilitate this coordination and this referral of the patient.” (Individual interview).* *“We had to do a test on the children (…) this wait and this anxiety thinking that my children might have (…)*,* the wait for the kids*,* to see what they had*,* what they didn’t have*,* because my little boy was small and when he’d been playing*,* afterwards I’d touch him to see if he was sweating*,* and he was sweating*,* but the gene is inside.” (Individual interview).* *“I’ve been on the waiting list for assisted reproduction (…) for almost three years. When it was finally my turn*,* they said no. (…) I’ve given up.” (Individual interview).*

### Experience with the disease

#### Symptomatology and disease progression

The symptomatology and progress of the disease vary significantly from patient to patient and depend on the years diagnosed as well as the time taken to receive the diagnosis. Some of the first symptoms experienced include pain, fever, burning in the hands and feet, fatigue, kidney problems, gastrointestinal problems, lack of sweating, balance problems, angiokeratomas, swelling of the hands and feet, heart conditions, migraines, and difficulty sleeping, among others. Some patients say that they have suffered mini strokes and cognitive impairment, specifically affecting processing speed, attention, and memory, although they do not know whether these are related to Fabry disease.

The presence of symptoms since childhood made some participants normalise this situation, remembering similar symptoms in their already deceased relatives. Sometimes, the presence of other conditions or health problems led to different tests being carried out, which incidentally helped the diagnosis to be reached, and in turn facilitating the initiation of diagnostic tests in other relatives. On some occasions, erroneous diagnoses were received that had a high emotional impact.

#### Medical specialists visited

Until they were diagnosed with Fabry disease, the participants went to see several specialists, and these consultations with the doctor were focused on the specific symptoms that they suffered at the beginning. Initial contact was made with Primary Health Care, then they were referred to specialties such as nephrology, cardiology, ophthalmology, neurology and dermatology, as new symptoms appeared that had to be assessed by other specialists. In this respect, when diagnosed, the participants say that they received little information from specialists about the disease.

With regards to the support received from doctors when diagnosed, most participants describe a shortage or absence of expert professionals, particularly a few years ago. In some cases, the feeling of a lack of coordination among medical professionals is also noticed.

All the participants say that they underwent a family screening once the disease had been diagnosed, which meant it could be detected more easily in younger members of their respective families.

#### Emotional impact

The diagnosis of Fabry disease has a significant emotional impact on both the health of any individual suffering from it as well as that of their loved ones, and it is also a difficult disease to understand. This lack of social understanding is highlighted by the patients interviewed as one of the main challenges that they face.

In general, patients report that the disease has a great emotional impact, which usually manifests itself with the presence of anxious-depressive symptoms, including nervousness, apathy, anhedonia, a lack of pleasure, and a feeling of emptiness. In relation to the symptomatology associated with the disease, the participants express their anger and frustration at the interference of pain, concern about the problems associated with vital organs, problems sleeping, fear of the consequences of the disease on their relatives, concern about the evolution of the disease, and fear of dying. In addition, feelings of restlessness, worry, hopelessness, uncertainty, acceptance, and resignation are described before the diagnosis with a view to the future, and the genetic character of the disease also affects their close relatives.

Throughout the interviews, feelings of guilt generated both internally by the participants’ own thoughts and externally by other people, including some medical interactions, are observed. These feelings are accompanied by stigmatisation, shame, frustration, loneliness, and helplessness due to a lack of information and resources. These situations, which require a significant emotional management from the patients, in turn cause relationships with family, partners, and other people to be affected.

#### Resources used to cope with the disease

With regard to the resources used by patients to better cope with the disease, the participants give a wide variety of responses: pharmacological treatment, support from patient associations, family and friends, a team of trustworthy health professionals, and acceptance of the disease along with a positive attitude. In that sense, for all participants, patient associations are a highly valued resource that provide emotional support and valuable benefits for patients. It is an opportunity to share experiences and interact with other patients, which makes the participants feel safe, supported, and at ease, especially in the early stages of the disease after receiving the diagnosis, avoiding the feeling of loneliness that is often experienced. In addition, belonging to a group makes it possible to find out about, accept, and go more deeply into the disease, its evolution and its complications, since its members are at different vital moments, allowing them to share experiences and coping strategies.

In relation to the coping strategies perceived as positive by the participants, the most prominent ones are acceptance, assimilation, normalisation, the fighting spirit, hope, and relief from the absence of symptoms or from receiving the diagnosis. Seeking social support through patient associations as a source of information, support, and peace of mind by having a social network is also highlighted.

Some patients implement planning strategies and search for possible ways to improve some symptoms, such as changing their diet or leading healthy lifestyles. They also use day-to-day normalisation strategies despite having to face work, social, and leisure limitations.

### Impact on daily activities

In general, patients look for social and leisure activities that they can take part in, trying to adapt them based on manifestations of the disease (e.g., fatigue, heart or kidney problems, diarrhoea, etc.), seeking alternatives to be able to integrate into their social network. It is often mentioned that they have to avoid or cut down on certain types of activities and also refrain from unhealthy behaviours (e.g., alcohol consumption at parties).

Among the sports activities carried out, hiking at an adapted pace is popular, as explained by people who take part in the activity. Less often, the depressive emotional state of participants can lead to demotivation when taking part in social and leisure activities.

In specific areas, such as the educational system, families of children with the disease stress that it is important to inform schools themselves so that both teachers and other students understand the disease and its consequences, especially regarding the impact on physical activity.

### Impact on work activities

Generally, for participants in the study, the disease has had a negative impact on their jobs, which is sometimes linked to the difficulty they face balancing work and the complications of the disease, such as pain, intestinal problems, tiredness, and fever. They say that being absent from work in order to go for treatment or to medical appointments is a complication that is also associated with the disease. The cognitive impact of the disease on work activity is also mentioned, especially slowness and lack of concentration.

In other cases, patients need to stop working and apply for disability benefits. Some are also affected emotionally and suffer symptoms of depression as a result of losing their job or changing their employment status (changing to a job that is more flexible, retirement, disability benefits, and even dismissals). Some patients demand a better understanding in the workplace; they say that they have experienced situations in which their colleagues have questioned their disease, showing little empathy, and that their situation is not understood either when they apply for disability benefits.

Some of the participants state that their companies are flexible and adapt to their needs, for example, by avoiding carrying out certain activities at specific times so as not to exacerbate symptoms or by offering the possibility to change shifts.

### Experience of the family environment

In relation to the emotional impact of the environment, the families of the interviewees have to go through an adjustment process in light of the significant psychological impact brought about by the disease.

Likewise, there is a shift in some expectations, and at times, there is a change in family roles where, for example, the carer becomes the relative who is cared for. Furthermore, conflict situations may arise in the doctor-family relationship, with blame being put on the behavior of concerned families, especially with respect to child patients. It should be noted that the majority of patients receive mutual support from their families, especially when more cases of the condition are detected within the same family.

Regarding the parenthood issue, the interviewees show great concern when thinking about having children. On the other hand, parents who receive the diagnosis after the birth of their children describe waiting for the results of their children’s genetic tests with great anxiety.

It should be noted that the majority of patients receive mutual support from their families, especially when more cases of the condition are detected within the same family. However, several of the interviewees say the opposite with regard to noticing a lack of understanding and support from the people around them, as others don’t fully understand the disease, with this situation in some cases leading to divorce.

Family support is given via different resources, such as emotional expression and support, religiousness, and optimism. Several of the interviewees report the opposite in reference to the fact that they perceive a lack of understanding and support from the environment, since others do not fully understand the disease.

The interviewees have developed a fighting spirit with the aim of not breaking the family balance. They refer to the need for understanding and patience regarding their illness and its consequences on the part of their relatives and the social environment. However, there are families that turn Fabry disease into a taboo topic, with an avoidance and denial towards emotional expression being observed in relation to said topic and even the impact on the family being blamed.

Regarding sexual relationships, most of the participants in the study say that these have been affected as a result of the disease, as fatigue and other symptoms mean they have sex less often and, consequently, this creates distance between couples (in some cases, the situation causes them to break up). Difficulties linked to the disease tend to include erection problems, loss of libido, feelings of inferiority, low self-esteem, and a low mood.

### Experience with pharmacological treatment

Regarding pharmacological treatments, patients express that there are access difficulties depending on the community of residence. They report that it is necessary to be able to access treatment from the moment of diagnosis and that the home treatment administration service is not available in all hospitals.

All patients taking part in the study state that they have had a good or very good experience with enzyme replacement therapy, associating it with benefits and delayed symptoms. However, they do not see great improvements in their symptoms when there is already a high disease impact.

In general, patients prefer to receive the treatment at home, as it is more convenient and can be adapted to their work. However, some participants express concern because, in certain cases, this home administration service will be taken away from them without any alternatives being offered.

Participants who receive treatment at the hospital mention that each time the treatment is administered, it takes up at least between three and five hours of their time (one and a half hours to prepare and administer the treatment, plus approximately two or three hours of travelling, depending on the distance between their home and the hospital).

Despite this preference, the experience at the hospital is good, and they welcome having the chance to express themselves and receive emotional support as they are in contact with other patients. There are also patients who prefer to go to the hospital to receive treatment because, if any complications were to occur, they believe they are safer.

### Team of health professionals

The professionals with whom adult patients have most contact are nephrologists, internists, and cardiologists. Other specialties to which they are referred are neurology, otorhinolaryngology, ophthalmology, dermatology and urology. However, only half of the participants seem satisfied with their medical team, mentioning a lack of communication and information, little empathy, limited specialised information, and coordination among professionals, as well as a shortage of training with respect to some doctors.

Those who do seem to be satisfied with their medical team mention good communication and a very close relationship, which shows that they are interested in the patient; this is also seen outside consultations, as patients are monitored well and are able to settle any doubts, where necessary.

Support from psychologists or psychiatrists is important in order to deal with the emotional impact of the disease. Nonetheless, the majority of interviewees say that they have been forced to look for other specialists in the private sector, particularly in the case of mental health professionals.

In this regard, the main sources of information about the condition for patients are doctors of reference, patient associations, social networks (such as Facebook and Instagram), Google, and conferences for patients and professionals. As for content, the participants look for general information about the disease (symptoms, treatments, and life expectancy, for example) and about diet, sexuality, motherhood, testimonies from other patients, etc. They have no preference regarding the format, but they do appreciate clear and concise information that is prepared by professionals.

### Unmet support needs

Patients with Fabry disease show various unmet needs, some of which are often mentioned in interviews. One of these is for the general public to have a greater knowledge of the disease and the repercussions that it has on the day-to-day lives of patients. The need mentioned most by all the participants is to have more information, support, and understanding from people around them and society, improving empathy and raising awareness about the difficulties faced by people with Fabry disease while giving it visibility.

In the same way, this misunderstanding also affects the workplace, where they believe that help is needed to apply for disability benefits. Some patients say that it should be easier for them to get an early retirement. Particular attention is also drawn to the need for professionals who are responsible for assessing disabilities to have a better knowledge of the condition.

In terms of the environment’s ignorance of the disease, the participants consider it important to carry out activities to make the disease visible and raise awareness about it in society. This is essential to obtain greater public awareness and also so that some people can feel identified because their symptomatology is similar and the disease can be diagnosed early.

The speed of diagnosis from the first symptoms is described as another unmet need, so protocols are created as soon as primary care physicians observe any symptoms in order to rule out the disease or start treatment as soon as possible. This need is linked to the perception of a lack of follow-up by the medical team and the need for training and coordination among the specialists looking after patients. They say that sometimes it has to be the patients themselves who inform doctors about Fabry disease, which sometimes causes health professionals to give negative and rejection responses.

The participants believe that there should be better access to treatments, that home administration should be guaranteed in all regions, that research on this condition should be increased, that it should be diagnosed more quickly, and that there should be better coordination among specialists.

When the participants in the study are asked about possible additional private support resources, the service most in demand is psychological support for both patients and their families, as the latter also need help in order to understand what this disease is and how it progresses, as well as their experiences from the point of view of the ill person, which makes it easier for them to empathise with their situation.

It is also considered important to have information on nutrition and dietary recommendations according to the disease. Likewise, specialised care in physiotherapy is deemed to be another need for these patients. As the disease progresses, there is a need for healthcare resources that include support, as well as specialised centers. The patient representative points to the geneticist as a fundamental specialist who should coordinate the process from the detection of the disease, although in practice this is not yet a reality.

Among the main needs identified by the patient representative, the deficiency in the coordination and referral of patients stands out, especially in cases where patients live in rural areas or are far from the health centers that can offer them the necessary care. The need to train professionals is also mentioned. Furthermore, the representative explains the need for a specific patient registry in order to know the actual number of patients. He also refers to the fact that it would be necessary for professionals who are responsible for assessing disabilities to have a better knowledge of the condition, as well as to include Fabry disease in newborn screening.

In relation to parenthood, the interviewees show great concern when thinking about having children, and they anxiously await the results of the genetic tests of their children. Some patients have made the decision not to have children due to the disease, while others have chosen to adopt or use assisted reproduction techniques, drawing particular attention to the shortage of public funding.

## Discussion

A qualitative and transversal study was carried out with FD patients, relatives, and one representative of the local patient association, with the main objective of examining in depth the quality of life and unmet needs of patients diagnosed with FD in Spain in relation to their disease and treatment. Even if the majority of studies involving patients diagnosed with Fabry disease evaluate quality of life using quantitative measures and standardised scales, complementing this knowledge with the use of qualitative methodologies would help to further explore the specific needs and concerns of this group of patients, their relatives, and, consequently, the socio-health agents involved in the care administered for this disease.

One of the findings of the study is that the symptoms and evolution of the disease are highly variable among the patients interviewed and depend on the years diagnosed as well as the time taken to receive the diagnosis. Until they were diagnosed with Fabry disease, the participants went to see several specialists. The diagnostic process, or diagnostic journey, is a painful experience in most cases. Only half of the participants are satisfied with their medical team, while the others expressed their discomfort due to the lack of communication, little empathy, limited information and coordination between professionals, as well as the lack of training of some doctors. Most of the interviewees have been forced to look for other specialists in the private sector, especially in the case of mental health professionals. The scarcity and/or poor communication of health professionals and their inability to provide patients with a comprehensive prognosis are a constant source of concern, frustration, and disappointment for both the patients themselves and their families [19, 20].

Diagnosis is a moment of great impact, both for one’s own health and for that of loved ones, and it is also a disease that is difficult for others to understand. The patients reported that the disease entails a great emotional shock that usually manifests itself with the presence of anxious-depressive symptoms that include everything from apathy, loneliness, guilt, frustration, and sleep problems. The identification and diagnosis of a rare disease not only have important psychological consequences for patients and their families, but it is also key to starting the appropriate therapeutic recommendations as early as possible. Diagnosis provides important prognostic information, correct genetic counselling regarding recurrence risk, and predictive testing in relatives [21]. In this sense, it is advisable to improve the training of specialists and the communication between them. Interdisciplinary support strategies for the diagnosis of rare diseases must be promoted and translational research facilitated, which would allow a faster diagnosis of patients and guarantee better care for this group [21].

Regarding the emotional impact of the environment, the families of the interviewees have to go through an adjustment process in light of the significant psychological impact brought about by the disease. These rare diseases have a considerable impact on the quality of life of not only patients but also of their families, who may notice a great impact on their social, professional, and family lives [22]. Likewise, there is a shift in some expectations, and at times, there is a change in family roles. This adjustment leads to a process of transformation in the person, materialising a change of life [23].

Some patients reported cognitive impairment, specifically affecting processing speed, attention, and memory, although they do not know whether these are related to Fabry disease. Fabry disease appears to be associated with cognitive impairment, specifically in executive functioning, information processing speed, and attention. The preserved domains are general intellectual functioning, memory, naming, perceptual functioning, and global cognitive functioning. In this sense, severe cerebrovascular disease, such as stroke or dementia, and psychological disorders such as depression can likely affect cognitive functioning in Fabry disease [24].

As described above, the main challenges that patients face are: understanding the disease, leading a normal life, social misunderstandings, as well as emotionally managing the disease. The scientific literature reflects how people with rare genetic health conditions feel socially excluded by friends and family [25]. Since people believe that they will suffer discrimination due to their illness or due to a lack of understanding of their condition, sometimes combined with health-related stigma, patients can adopt strategies that minimise the risk of rejection, such as keeping their health status a secret [26]. It seems necessary to carry out actions to raise awareness of the disease that are aimed at the general public, the patients themselves, and the people around them, focused on giving it visibility by means of patient testimonies that describe the reality of their everyday lives with Fabry disease.

Other challenges that the patients had to face were related to social, work, and educational fields, in addition to relationships with family and partners. Similar results have been found in previous studies with people with rare diseases [25]. The fact that Fabry is an “invisible” disease to the normal eye makes it more difficult for general people to understand its impact and complications for the patient. In specific areas, such as the educational system, families of children with the disease stress that it is important to establish communication channels with schools and to sensitise both teachers and other students about the disease and its consequences, especially in relation to physical activity [16, 25]. In terms of partners, most of the participants in the study report that their sexual relationships have been affected as a result of the disease. Sexual well-being is not adequately addressed with people with a rare disease, and it is an issue that affects the quality of life and well-being of the affected people and relationships with partners [16, 27].

In relation to parenthood, the interviewees show great concern when thinking about having children, and they anxiously await the results of the genetic tests of their children. In that sense, the role of genetic counselling is essential to providing information and personalised support to patients with rare diseases. However, the current situation of genetic counselling for rare diseases in our context is jeopardized by the lack of recognition of Clinical Genetics as a healthcare specialty within the national health system [28]. Additionally, it would be important to provide information and guidance on assisted reproduction techniques for these patients so that they can have better resources and high-quality information about family planning.

Underlining some of the aspects mentioned above, the patients especially highlighted various unmet needs with respect to their illness, which included: increasing the speed of diagnosis; greater training of specialists; having access to treatment from the moment of diagnosis; the creation of a single patient registry; and greater knowledge of the disease in society, among others. These issues and similar ones have been widely debated as primary needs to be addressed within European policies and plans in order to ensure that patients with rare diseases have access to high-quality care, including diagnostics and treatments for those living with the disease [29–32].

Regarding the resources used by patients to better cope with the disease, the participants give a wide variety of responses: pharmacological treatment, family and friends, a team of trusted health professionals, and acceptance of the disease with a positive attitude. Unanimously, patient associations are a much-appreciated resource that offers emotional support and invaluable tools to patients. It is an opportunity to share experiences, interact with other patients, and make them feel safe, supported, and at ease. Patient organisations can often be the only accessible source of up-to-date and reliable information on the disease. They also offer essential support to patients, especially those who have recently been diagnosed, by guiding them through the healthcare system and giving them someone to talk to [19]. It is advisable to facilitate access to mental health professionals for patients and their families, as psychological support is the service that is most in demand for the participants.

Regarding the experience of patients with pharmacological treatment, all patients taking part in the study state that they have had a good or very good experience with enzyme replacement therapy, associating it with benefits and delayed symptoms. However, it is important to note that patients express that there are access difficulties to treatment depending on the community of residence. According to the 2022 AELMHU access report, of the total 146 orphan drugs with marketing authorization in the EU, only 43% of them (63) are financed by the SNS in Spain. In addition, of the 63 financed drugs, 48% (30) have restrictions, that is, limitation on indications or unfunded indications [33].

In general, patients show a preference for home administration of the treatment, as it allows them to better conciliate the treatment with their work lives. They consider the necessity of accessing treatment from the moment of diagnosis and that the home treatment administration service would be available in all hospitals. However, some participants are concerned because, in some cases, this service will be withdrawn without being offered alternatives. Those who receive medication in the hospital mention that each administration takes at least 3–5 h of their time, which significantly impacts their personal and work lives. However, the experience in the hospital is good and they positively value having the opportunity to express themselves, to have emotional support by being in contact with other patients, as well as the possibility of being assisted in case potential complications occur [34].

Although this study describes the needs and perceptions of patients and relatives with Fabry disease in detail, it must be considered that it also has a series of limitations. First of all, this study focuses exclusively on a qualitative methodology, and we suggest that future studies use a mixed methodology, applying validated questionnaires that support qualitative measures, including general and specific questionnaires. Other limitation of the study was the small sample size. In terms of the sociodemographic characteristics of the sample, this study has not considered the different classic or non-classic forms of the disease, severity, or genotype. Gender has been considered so that both men and women are represented, although this variable could be further explored.

The findings of this study must be considered as knowledge to be taken into account in clinical practice as well as in legislative measures that promote policies in order to bring about a better quality of life for this group of patients. The themes described throughout the study can also be used by other professionals and scientists when applying intervention programmes, taking the specific needs of this disease into account. On the other hand, it involves the acquisition of knowledge that may be useful for the development or improvement of the few quantitative assessment questionnaires regarding quality of life specific to Fabry disease, as well as measurement of the patient-reported outcomes and experience of health. These specific measures are also essential in order to efficiently assess evaluation and intervention strategies from the perspective of quality of life as well as cost-benefit analysis.

Patients with Fabry disease and their relatives are forced to deal with a condition that affects their quality of life, including associated symptoms, the diagnostic process, medical care, treatment, and the course of the disease. In addition, patients perceive a lack of understanding from the social environment, including work and educational areas, among others. The results of this study draw attention to the need to gain more knowledge of this disease as well as to provide support resources based on the needs of these patients, including access to psychological professional support, which will help improve their quality of life and promote disease management and treatment.

## Electronic supplementary material

Below is the link to the electronic supplementary material.

Supplementary Material 1

## Data Availability

The datasets generated and/or analyzed during the current study are not publicly available due to the database containing confidential information, but are available from the corresponding author upon reasonable request. In fact, the vast majority of the testimonies of the participants are included in the article.
