# Supplementary material for: Quality of life and unmet needs in patients with fabry disease: a qualitative study
Source: Orphanet J Rare Dis. 2024 Oct 18;19:389. doi: 10.1186/s13023-024-03412-6 (PMC11490088; doi:10.1186/s13023-024-03412-6)
Supplement: Supplementary file 1 — Supplementary Material 1 [file 13023_2024_3412_MOESM1_ESM.docx]

| Reviewer #1: Thanks for submitting this peice of work.  1. Several grammar mistakes were noted. I suggest editing the text as per the attached file.  2. High risk of plagiarism was detected via different platforms. I suggest submit your paper for plagiarism check using one of the available platforms. | Thank you very much for reading the work and for the recommendations for improvement.  The level of plagiarism of the text has been reviewed. Many of the coincidences are related to definitions and concepts that cannot be modified. The current plagiarism level is correct (9%-Turnitin).  Likewise, possible grammatical errors have been reviewed. |
| --- | --- |
| Reviewer #3: This articles describes an in-depth assessment of quality of life of Fabry patients in Spain by use of semi-structured interviews and focus groups comprising patients, family members and patient organisation representatives. The results section describes the typical experiences of the interviewed patients with Fabry disease, in medical, work, social and family settings. The insights of the patients interviewed will be of interest to anyone involved in establishing rare disease clinics and networks, to researchers wishing to conduct clinical trials in Fabry disease patients, to patient organisations and even to patients and families, as the findings are written in a very clear and readily understandable way. The main conclusions of the paper - that psychological support, better communication and co-ordination from the medical teams treating patients, and for more educational resources about Fabry disease are likely to be applicable outside Spain, and will be of  most benefit to clinicians treating Fabry patients. There is sufficient novelty and interest, the conclusions seem well supported, and the limitations of the study are mostly well addressed. I recommend acceptance following minor revisions: | Thank you very much for reading the work, for the feedback and for the recommendations for improvement. |
| Page 4 - definitions of the medical terms used e.g. anhidrosis would be beneficial to non-clinician readers  Page 4, paragraph 3 - reference 9 referred to twice | Definitions of the medical terms used have been included.  The twice reference has been eliminated. |
| Page 5, paragraph 2 - use of the word 'guarantee' is too strong. Given the small sample size, it is impossible to guarantee that the views of the patients selected can be generalised to the entirety of Fabry patients and families. Replace 'guarantee' with 'to attempt to capture the diversity of experiences of Fabry patients' or similar.  Page 6, paragraph 2, last sentence - change 'focus groups' to 'focus group'.  Page 6-7 - sentence 'In addition, the expressions and comments in free format of the participants were taken into account as additional data to the questions of the semi-structured interviews, thus considering the negative cases in comparison with the most common cases.' - it is not clear what this sentence means.  Small sample size should be added as a limitation of the study. | Ok, the phrases have been modified. |
| The discussion is over long, and includes a lot of repetition from the results e.g. the impact on patients in leisure and work situations - an effort should be made to make the discussion more concise. | Because the qualitative approach takes into account a multitude of variables, it is difficult to carry out a concise discussion. However, an attempt has been made to be more precise. The discusión has been modified. |
